# Economic Analysis of National Program for Hepatitis C Elimination, Israel, 2023^1^

**DOI:** 10.3201/eid3010.240210

**Published:** 2024-10

**Authors:** Yuval Dadon, Francis B. Mimouni, Ariella Toren, Tal Morgenstern, Lior Barak, Joseph Mendlovic

**Affiliations:** Ministry of Health, Jerusalem, Israel (Y. Dadon, A. Toren, T. Morgenstern, L. Barak, J. Mendlovic);; Leumit Health Care, Tel Aviv, Israel (F.B. Mimouni);; Sackler School of Medicine, Tel Aviv (F.B. Mimouni);; Shaare Zedek Medical Center, Hadassah-Hebrew University School of Medicine, Jerusalem (J. Mendlovic)

**Keywords:** Hepatitis C, viruses, elimination program, cost estimation, Israel

## Abstract

In 2021, the Israel Ministry of Health began a national hepatitis C elimination program. Implementing a World Health Organization goal, Israel’s program involved targeted screening, barrier minimization, workup simplification, awareness campaigns, and a patient registry. We evaluated program costs for testing and treatment. By May 15, 2023, the program had identified 865,382 at-risk persons, of whom 555,083 (64.3%) were serologically screened for hepatitis C virus (HCV), which was detected in 24,361 (4.4%). Among 20,928 serologically positive patients, viremia was detected in 13,379 (63.9%), of whom 10,711 (80%) were treated, and 4,618 (96.5%) of 4,786 persons receiving posttreatment HCV RNA testing had sustained virologic response. We estimated costs of ₪14,426 (new Israel shekel; ≈$3,606 USD) per person whose HCV infection was diagnosed and successfully treated. The program yielded screening and treatment in almost two thirds of the identified at-risk population. Although not eliminated, HCV prevalence will likely decrease substantially by the 2030 target.

Active hepatitis C virus (HCV) prevalence (i.e., HCV PCR positivity) in Israel is estimated to range from 0.1% to >5.5%, depending on risk group affiliation; seroprevalence (i.e., HCV antibody positivity) among the general population is ≈1.96% (*1*–*3*). In Israel, risk groups include immigrants from the former Soviet Union; persons who received blood transfusion before 1992, when HCV testing for blood donations was initiated; persons who inject drugs (PWID); persons with other bloodborne diseases, such as HIV or hepatitis B; and persons who have undergone invasive procedures in places lacking universal precautions to bloodborne infections (*1*,*4*). Untreated HCV infection spontaneously resolves within 6 months postinfection in ≈25% of patients (*5*), but HCV can continue latently for years until complications such as liver cirrhosis or carcinoma emerge (*6*). In addition, chronic HCV carriers remain contagious.

The availability of highly effective direct-acting antiviral (DAA) medications enabled a sustained virologic response (SVR) in >95% of cases, prompting the World Health Organization (WHO) to declare in 2016 that the goal of eliminating HCV by 2030 was achievable (*7*). Subsequently, Israel’s Ministry of Health (MoH) launched an HCV elimination program in 2021 (*8*).

We hypothesized that a targeted approach to HCV elimination enabled detection of active disease in >5% of high-risk persons. In addition, we believed that a systematic program could lead to more carrier detection and substantially increase the number of patients treated over time. Finally, we hypothesized that the SVR rate exceeded 95% in compliant patients. Thus, we aimed to evaluate the costs of the HCV elimination program, assess program efficacy in terms of SVR in treated patients, and develop policies for improving program compliance and maximizing SVR.

## Methods

### Program Elements

The Israel MoH used 5 principles to guide development of the HCV elimination program. Those 5 principles were targeted screening guidelines; identification of barriers and development of strategies to minimize those barriers; clinical workup simplification; awareness campaigns; and a national patient registry. 

#### Targeted Screening Guidelines

In 2021, MoH implemented new directives mandating that every citizen who emigrated from an endemic country or identified in any of the risk groups be referred for HCV antibody screening testing. Screening was conducted by each of the 4 health maintenance organizations (HMO), the Health System of the Israeli Defense Forces (IDF) for drafted soldiers, and the Israeli Prison Services (IPS) for long-term prisoners and incomers. MoH also deployed dedicated microelimination taskforces for subpopulations that did not regularly visit a primary care physician (PCP) and that required proactive outreach, such as PWID. Subsequently, all serologically positive persons with a positive reflex PCR test indicative of viremia were started on DAA treatments, as dictated by the Healthcare Basket, the funding system for healthcare in Israel.

#### Identification and Minimization of Barriers

The MOH developed a taskforce networking policy to assure ongoing collaboration between all relevant parties. Networking involved extensive discussions with all HMOs, IPS, IDF, physician and patient union representatives, the National Councils, and the public. All taskforce delegates were asked to identify potential challenges in program materialization and to offer tailored solutions. Recognized challenges included at-risk population identification, treatment costs, workup and testing costs, and clinic availability.

To address challenges in identifying at-risk populations, when an HMO identified persons with possible inaccurate birth country data, MoH assisted in data retrieval from the national citizen registry. Those data were later transcribed into the well-established HMO electronic medical record (EMR) system. The EMR could automatically alert healthcare workers of persons at high risk for HCV, advise on HCV risks, and refer those patients for testing.

HCV treatment cost gradually extended over time to include a wide range of indications for all disease severity and genotyping (Appendix). Thus, every HCV patient in Israel eventually was eligible for free treatment.

Workup and testing costs were minimized by cancelling the requirement of genotyping assessment because pan-genotypic DAA medications were available (*9*). In addition, costly measures for fibrosis assessment, such as imaging-based diagnostic testing, were replaced by serum laboratory parameter–based assessments.

**Figure 1:**
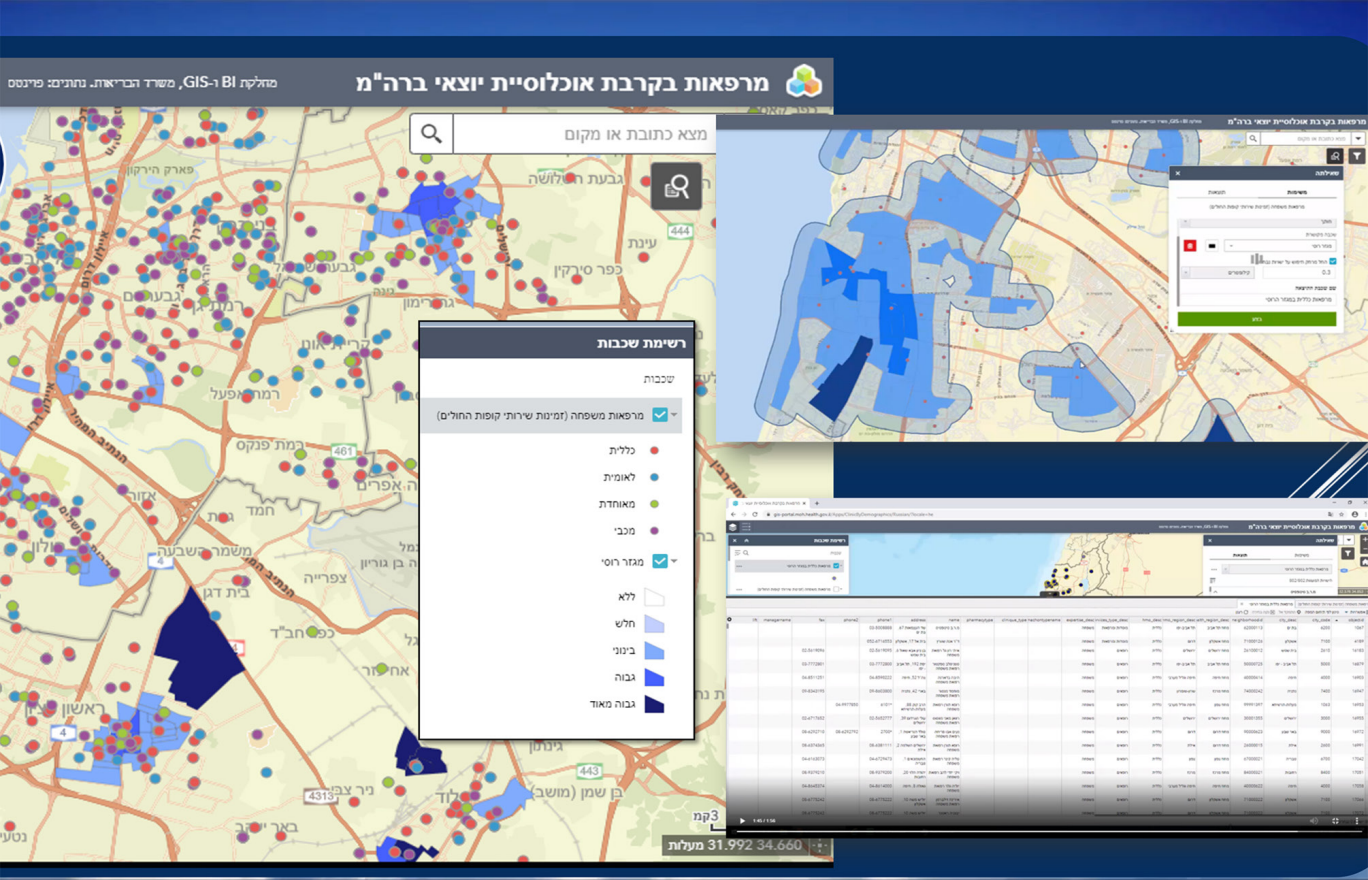
Geographic mapping in an economic analysis of a national program for hepatitis C elimination, Israel, 2023. Screenshot show Hebrew-language mapping by Points Location Intelligence company (https://points.co.il) in ArcGIS (www.arcgis.com). Upper right inset shows clinic locations (orange dots) at the beginning of the program. Blue shading indicates areas with higher populations of immigrants from the former Soviet Union, who are at higher risk for hepatitis C virus infection; intensity of shading increases as population numbers rise. Detail on left shows increased clinical sites added as part of the hepatitis C elimination program. Each colored dot (orange, blue, green, purple) represents a clinic and its affiliation to 1 of the 4 health maintenance organizations, shaded areas that represent the prevalence of population at risk in a given geographic area. Darker blue indicates higher concentrations of at-risk populations. Combining the 2 parameters (clinics distribution and density of population at risk density) enabled a systematic strategy for a targeted approach to identify and list clinics for higher yield in program accomplishment.

To address clinic availability, MOH used geographic information systems to map areas of high-risk populations. Using those maps, HMO clinic distribution was adjusted to address targeted populations (Figure 1).

#### Clinical Workup Simplification

Subsequent workup for patients with HCV viremia was minimized to include only FIB-4 classification assessment, a blood-based diagnostic test for liver fibrosis that relies on 4 parameters: alanine aminotransferase, aspartate aminotransferase, platelet count, and patient age (*10*). FIB-4 eliminated the need for fibrosis and genotyping assessment via time-consuming measures. Use of FIB-4 was based on current clinical guidelines and availability of DAA drugs that do not require genotyping (*11*). FIB-4 reduced the burden on persons with a diagnosed HCV infection and enabled rapid treatment initiation.

FIB-4 index of 1.4 was the defined cutoff for patients with mild disease; those patients could be treated by their PCP and then have HCV RNA PCR testing 12 weeks after treatment. Severe cases (i.e., FIB-4 >1.4) were referred for gastroenterology evaluation, which previously was used for all HCV-positive cases, regardless of severity (*12*).

#### Awareness Campaigns

MoH provided healthcare workers with online e-tutorials on the HCV elimination program and their role in it. MOH and HMOs distributed leaflets on HCV to the public in all relevant languages. Leaflets described the HCV asymptomatic phase and the need for early diagnosis and treatment. In addition, MoH opened an HCV web portal (https://govextra.gov.il/ministry-of-health/hepatitis/c-en) and uploaded a questions and answers video to the MOH YouTube channel https://www.youtube.com/watch?v=9Z8vivI0kqA). The strategic network distributed links to the public. MoH representatives also executed media interviews, advertisements, and awareness campaigns. Finally, MoH collaborated with municipalities, unions, and other ministries (e.g., immigration and social affairs) as a major component for increasing awareness and compliance to encourage citizens to undergo HCV screening.

#### National Patient Registry

The status of each identified patient was monitored via a national registry reported to the MoH in an individualized, yet anonymized, format. Registry data included patient’s status from the initial serology testing through subsequent PCR testing, treatment, and eventually SVR, where indicated.

### Program Cost Estimation

Annual healthcare costs in Israel are mostly financed by MoH via Healthcare Basket. All citizens have mandatory coverage through 1 of the 4 available HMOs. Coverage comprises all medications, tests, and services that HMOs are obligated to provide to insured patients. 

Each year, a joint government–HMO committee examines the scope and costs of the Healthcare Basket. The committee recommends an annual cost expansion by adding new medical treatments to the existing budget of the previous year on the basis of an estimation of the number of eligible patients and the cost of the new treatment. Each HMO is granted funds proportional to the number of enrolled patients. The entire HCV elimination program, including healthcare workforce, testing, and treatment, is provided at no cost to all HMO members. Although we do not provide details of the exact calculations, the costs estimated in this study likely reflect those agreed upon by finance specialists from both the government and the HMOs.

To assess the cost per resolved HCV case, we considered costs for laboratory testing, government budget to cover treatment costs, and costs for liver fibrosis testing. We considered laboratory testing costs throughout a patient’s workup process, including serologic screening, subsequent PCR testing, and PCR testing for SVR confirmation. We used MoH’s tariff to determine the cost of each laboratory test (*13*). We calculated cost for a subpopulation that had already been tested for HCV and for the subpopulation for which testing was pending. To calculate the number of required subsequent HCV PCR tests for serologically HCV-positive patients, we assumed the proportion of positive persons among the not-yet-tested subpopulation on the basis of the findings from the tested population. We then estimated overall costs using those assumptions.

We assessed the governmental budget to cover HCV treatment costs, including the gradual expansion of indications over the study period (*13*) (Appendix). Medication pricing represents the final price after MoH negotiated with pharmaceutical companies.

We also included testing for liver fibrosis via imaging or biomarker testing and genotyping for patients with viremia in the assessment. Those tests were later found to be redundant when DAA medications became available. DAA medications are effective against all HCV subtypes and require no prior testing; thus, the overall cost for liver fibrosis assessment was virtually exchanged to cover the cost of other supportive HMO promotional activities, also budgeted by MoH, as part of the Healthcare Basket.

We used available 2021 pricing to calculate all the costs and assumed that pricing changes were essentially unchanged throughout the observation period. We divided the sum of those elements by the number of all resolved HCV infections over the study period, as reported annually to MoH by the different HMOs.

### Statistical Analysis

We characterized and summarized at-risk persons by their workup and treatment stage at the country and HMO levels. At the HMO level, we calculated the number of persons in the target risk-group population who participated in the screening process; the prevalence of HCV positivity among persons serologically screened for HCV antibodies; and the prevalence of viremia among seropositive persons tested by PCR. We used χ^2^ tests to statistically assess differences between the rates of positivity among HMOs and considered p<0.001 statistically significant. We also reported HCV treatment over the study years and number of HCV patients treated on the state and HMO levels.

## Results

### Findings of Program Progress

#### Risk Group Identification

By June 13, 2022, the national registry identified a total of 865,382 persons at risk for HCV infection and their birth countries. Among at-risk persons, 98% (863,909) were actively insured by 1 of the 4 HMOs, and the other 2% were either at IPS or IDF or had emigrated out of Israel. At-risk persons included 26,004 persons who immigrated to Israel from Russia and Ukraine during the immigration wave of 2022 due to the escalation of the Russo-Ukrainian War. 

We classified immigration data by HMO affiliation. The 2 largest HMOs in Israel had the largest percentage of immigrants, 40.6% and 37.6% (Table 1). We noted no major differences in origin country or age-group distribution when comparing those distributions among HMOs.

**Table 1:** Distribution of identified risk groups by HMOs in an economic analysis of a national program for hepatitis C elimination, Israel, 2023*

HMO no.	At-risk persons†	Immigrants‡	Total at-risk persons	Screened for HCV	Serology			
					Testing pending	Positive	Seropositive + PCR-positive	% Viremia
1	73,023 (8.4)	859 (3.2)	73,882 (8.6)	51,891 (70.2)	21,991	2,396 (4.6)	748 (31)	1.4
2	113,982 (13.2)	1,680 (6.5)	114,426 (13.2)	50,387 (44.0)	64,039	1,545 (3.1)	235 (15)	0.5
3	318,380 (36.8)	6,621 (25.5)	325,001 (37.6)	163,386 (50.3)	161,615	9,795 (6.0)	5,614 (57)	3.4
4	359,997 (41.6)	16,844 (64.8)	350,600 (40.6)	289,419 (82.5)	61,181	10,625 (3.7)	6,782 (64)	2.3
Total	865,382	26,004	863,909	555,083 (64.3)	308,826 (35.7)	24,361 (4.4)	13,379 (54.9)	2.4

*Values were assessed by phase of screening status, prevalence of HCV serology and viremia in 4 HMOs. Values are no. (%) except as indicated. HCV, hepatitis C virus; HMO, health maintenance organization. †Data on at-risk persons included country of birth. ‡Persons who immigrated to Israel from Russia and Ukraine during the immigration wave of 2022 due to the Russia–Ukraine war.

#### HCV Screening Performance

**Figure 2:**
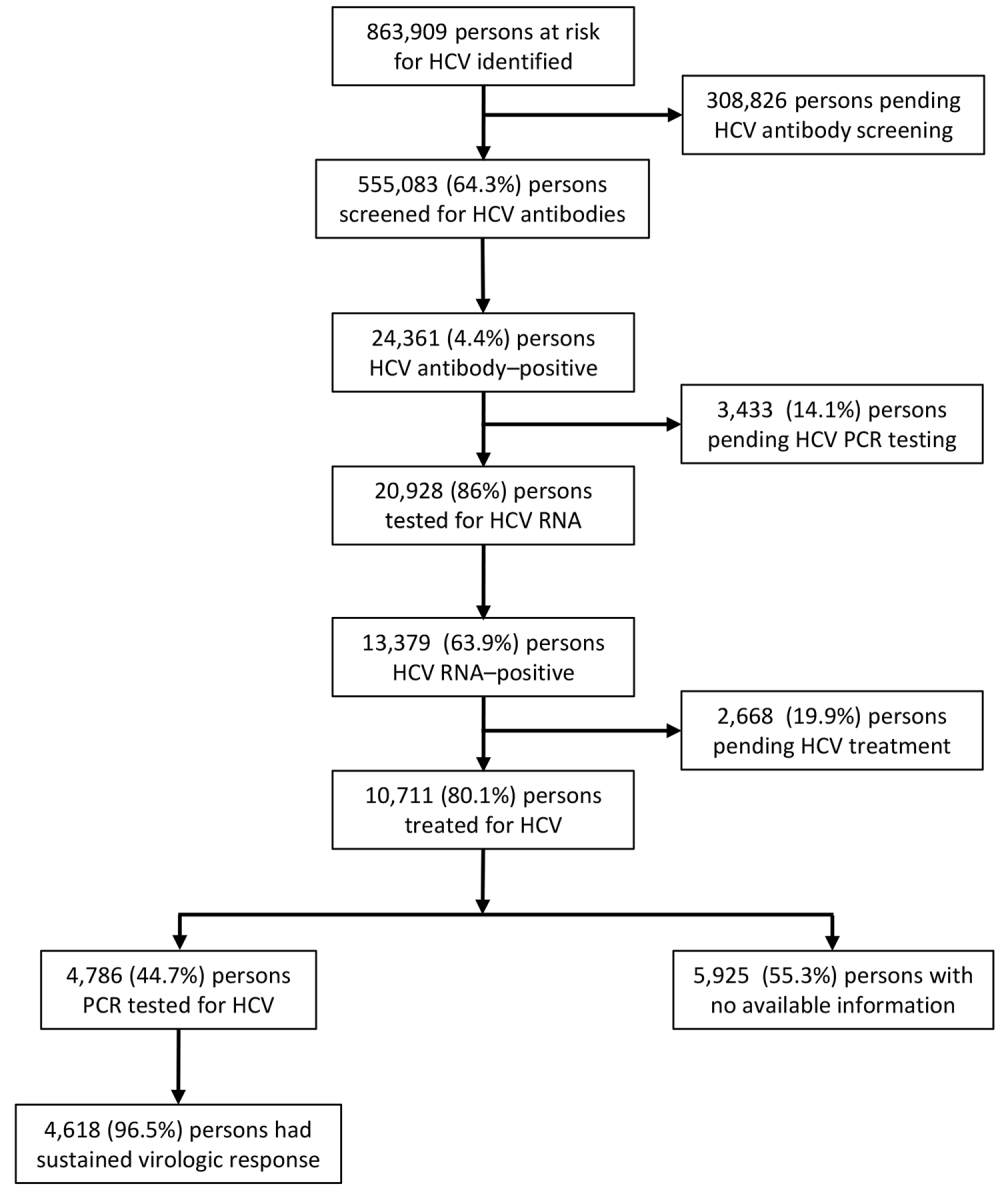
Flowchart of participant enrollment, virologic screening, and HCV treatment in an economic analysis of a national program for hepatitis C elimination, Israel, 2023. HCV, hepatitis C virus.

By May 15, 2023, a total of 555,083 (64.3%) persons in the target risk group population had already been screened for HCV (Figure 2). Screening occurred either through the national program or previously as part of the standard workup assessment per clinical requirement, such as liver enzyme elevation assessment.

#### HCV Seroprevalence

Overall, HCV seroprevalence was 4.4% among the 555,083 persons tested (i.e., 24,361 persons positive for previous infection) (Table 1; Figure 2). Seroprevalence ranged from 3.1% to 6.0% in the different HMOs (Table 1), and those differences were statistically significant (p<0.001).

**Figure 3:**
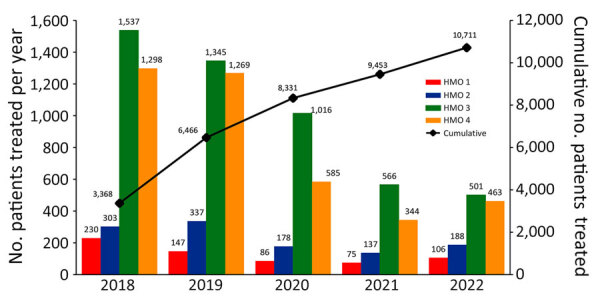
Assessment of patients treated in 4 HMOs in an economic analysis of a national program for hepatitis C elimination, Israel, 2023. Scales for the y-axes differ substantially to underscore patterns but do not permit direct comparisons. HMO, health maintenance organization.

Subsequent HCV PCR testing for 20,928 (86%) HCV antibody–positive patients identified 13,379 (63.9%) persons with an active HCV infection (Figure 1). The other 3,433 seropositive persons had not yet received further PCR testing, mostly because of noncompliance, despite HMO PCP reminders to get tested. DAA treatment was started and documented in 10,711 (80%) persons (Figure 3), according to MoH’s registry for drug prescriptions during 2018–2022.

#### SVR

Among 10,711 persons treated for HCV, 44.7% (4,786) had available data on HCV RNA testing 12 weeks after treatment completion, among whom 4,618 (96.5%) showed SVR. Patients who did not reach SVR as measured by posttreatment HCV RNA testing continued the standard care follow-up under their HMO, including repeated testing and follow-up. No data are available for that ongoing follow-up.

#### Pending Workup and Treatment

At the cutoff date for retrieved data, a total of 2,668 (19.9%) HCV patients had not yet started treatment. In addition, 14.1% (3,433) of serologically positive patients had not yet been tested for viremia.

### Estimation of Program Cost Per Resolved HCV Case

The overall cost per documented resolved HCV case was ₪14,426 (Israeli new shekel; ≈$3,606 USD). That estimation considers initial serology testing for the identified at-risk population, subsequent PCR testing for the seropositive patients, and PCR testing to confirm SVR (Table 2). The cost of the initial serology screening testing for the entire at-risk population of 863,909 identified persons was ₪20.3 million. PCR testing for all 24,361 seropositive patients was ₪10.61 million, and PCR testing for the 13,588 potentially seropositive patients was ₪5.92 million (assuming 4.4% positivity for HCV serology testing), which totaled ₪16.53 million for the entire at-risk population. However, in a worst-case scenario in which Israel could not test or treat additional persons, our assumptions show the country would spend ≈₪290.349 million to treat HCV, or ≈₪28,000 per resolved case. During the study period, the overall HCV treatment cost from the Healthcare Basket was ₪260.86 million (Appendix).

**Table 2:** Cost estimation per a resolved HCV case in an economic analysis of a national program for hepatitis C elimination, Israel, 2023*

Category	HCV antibody tested				Pending testing			Program total cost
	No. patients	Cost per patient	Total cost		No. patients	Cost per patient	Total cost	
Initial serology	555,083	23.5	13,044,451		308,826	23.5	7,257,411	20,301,862
PCR for positive serology	24,361	435.5	10,609,216		13,588†	435.5	5,917,724	16,526,939
Treatment								260,869,000‡
Posttreatment PCR for SVR	13,379	435.5	5,826,555		8,697§	435.5	3,787,544	9,614,099
No. SVR patients	12,911	NA	NA		8,392¶	NA	21,303	
Total program cost								307,311,900
Cost per resolved HCV case								14,426 ($3,606)#

*A total of 863,909 persons were evaluated via serologic testing for HCV antibodies. Costs are in Israeli new shekel, except where indicated. HCV, hepatitis C virus; NA, not applicable; SVR, sustained viral response. †Given 4.4% positivity for HCV serology tested. ‡Overall treatment cost at Health Basket. §Given 64% positivity for HCV PCR testing. ¶Given 96.5% HCV resolution. #Conversion to US dollars based on 2012 data from ExchangeRates.org (https://www.exchangerates.org). Data should be inflation adjusted for accumulated inflation during 1999–2021.

In the program, posttreatment PCR for SVR was performed for tested and potentially PCR-positive patients. Costs for that testing were ₪5.83 million for 13,379 HCV-confirmed and ₪3.79 million for 8,697 potentially PCR-positive patients, which assumes a 64% positivity for HCV PCR testing. Total costs for SVR testing were ₪9.61 million.

Our cost analysis assumptions might provide overly optimistic calculations. Indeed, by the analysis date, only 10,711 persons had started DAA treatment, and only 44.7% of those patients had data on posttreatment SVR. However, the cost analysis assumed that 21,303 persons would achieve SVR, which resulted in an estimated cost of ₪14,426 per resolved HCV case. For example, if 96.5% of persons treated with DAA medications achieved SVR, costs would be ≈₪30,000 per person with SVR.

## Discussion

Our data analysis shows that, within a 27-month period, Israel tested 555,083 (64.3%) of 863,909 persons at risk for HCV infection. The program aimed to identify all patients needing treatment by 2030, and our results indicate the program could be construed as a remarkable success. However, the program still has a long way to go to get as close to 100% as possible.

In addition, of the 555,083 persons tested, HCV seroprevalence was 4.4%, meaning 24,361 persons had positive results of previous infection. Subsequent PCR testing for 20,928 HCV seropositive persons yielded 13,379 (63.9%) with an active infection. Thus, 14.1% (3,433/24,361) seropositive persons had not yet received further PCR testing, despite PCP reminders to get tested. That 14.1% noncompliance rate is concerning because the PCR positivity rate among those persons may be much higher than the 64% positivity rate documented among compliant persons. Moreover, 80.1% (10,711/13,379) of persons received DAA treatment for HCV, indicating a treatment noncompliance rate of 19.9%. Furthermore, SVR was not evaluated in all treated patients, probably because eradication rates from DAA treatment are known to be extremely high. However, among 4,786 (44.7%) persons who received PCR tests, 3.5% still tested HCV RNA–positive.

Nevertheless, compared with other countries facing WHO’s initiative for 2030 HCV elimination, Israel shows great progress. A 2022 review of status of the HCV initiative in Europe showed that most countries are not on track to reach the WHO target (*14*). Some countries have not established policies or guidelines for treatment, prevention, or harm reduction. Moreover, because many existing national plans do not include wide-scale screening, HCV treatment rates have even been declining over the past few years. In contrast, in Egypt, a total of 49,630,319 (79.4%) persons out of a target population of 62.5 million spontaneously participated in screening during October 1, 2018–April 30, 2019 (*15*). In just over 7 months, 2.2 million HCV-seropositive persons in Egypt were referred for evaluation and treatment (*15*). 

From our data, in view of the lack of absolute compliance in serologic screening, HCV PCR testing, treatment completion, and post treatment testing, HCV likely will not be eradicated in Israel by 2030. Nonetheless, HCV prevalence likely will be substantially reduced, leading to a decrease in the number of new cases.

The program received heavy political support, which likely explains its sustainability. Estimated cost for HCV treatment in Israel gradually expanded by ≈2.63-fold over the years after initial inclusion of DAA medications in 2012. At that time, the overall aggregated Healthcare Basket allocation to treat HCV was ₪99.1 million a year. Allocations rose to ₪261 million in 2018, when all available HCV DAA medications were included and the indications for DAA use expanded to all disease severity and HCV subtypes (Appendix). Of note, policy makers were convinced of the value of investments in resolving HCV cases by 2016, when they included not only therapeutic agents but also ancillary diagnostic testing (i.e., laboratory and imaging for fibrosis assessment) that covered the additional ₪5.58 million per year in workup costs.

By enabling a risk-sharing model between the HMOs and pharmaceutical companies, MoH was able to provide no-cost testing and treatment to patients and later DAA medications free of charge. No-cost testing and treatment is a major factor driving adherence to the program. Also of note, the observed active HCV prevalence of 2.4% in the at-risk population in Israel seems to be lower than the expected prevalence of <5.5% for this subgroup based upon previous research (*1*–*3*). We suggest that HCV spontaneous recovery might be higher than 25% and might have increased over the years to >45.5% (*5*).

In 2020, the US Preventive Services Task Force issued a new recommendation for HCV screening, advising that all adults aged 18–79 in the United States without liver disease undergo screening for HCV (*15*). That updated screening recommendation expands the previous guideline, indicating screening only for persons born during 1945–1965 (*16*). Although Israel’s MoH did not adopt this recommendation, the results of our study could provide additional support for nonuniversal screening of adults in view of the higher degree of spontaneous recovery among infected persons. Nevertheless, additional investigation, such as a random sample of the general population, may be required to support a change in policy.

Our findings address the overall cost of Israel’s HCV elimination program, which is assumed to prevent long-term consequences among persons with resolved infection. Our findings require validation through epidemiologic investigations to verify the effects of the program on the incidence of HCV-related liver disease, residual chronic infection, and associated complications. Furthermore, additional research to accurately assess future cost savings and the impact on disability-adjusted life-years saved is warranted.

The first limitation of our study is that we based it on at-risk population testing and not on universal testing; thus, we cannot estimate the rate of seropositivity in the general population in Israel. Second, we did not have information on persons who emigrated from Israel; thus, our seropositivity rate could be underestimated. Third, completed data on treatment outcome and SVR status were lacking, partly because PCPs assume the high rate of resolution for DAA treatment might not warrant further PCR assessment; thus, SVR rates might be underestimated. Fourth, PWID are difficult for HMOs to follow, which might have contributed to noncompliance rates. Fifth, ≈36% of the at-risk population had not completed the initial screening despite HMO outreach attempts, and a fraction of those patients persistently refused to undergo any evaluation, although their motives for noncompliance are not completely clear. Thus, those patients, might be the most noncompliant, and might not have achieved HCV resolution even if treated. Sixth, the costs we report reflect costs for medications, tests, and scans going back to 1999 (Appendix). Those costs probably do not accurately reflect the cost to treat the active hepatitis C cohort identified through the elimination program, which formally initiated in 2021. In addition, the cost calculations provided here are estimates based on published information, such as pricing of medications or laboratory tests, and might differ from the real costs incurred by HMOs; thus, our treatment and testing costs could be underestimated. Finally, risk factors for HCV seropositivity, including age, sex, socioeconomic status, and area of residence, were not recorded in a comprehensive registry, as planned, because of operational limitations and the desire to initiate the program as soon as possible. Consequently, valuable analyses, such as differences between patients with and without viremia, could not be assessed and deserve further evaluation.

In conclusion, this study demonstrated that a targeted approach provided HCV screening to a large percentage (64.3%) of identified at-risk persons in Israel and a substantial steady increase in the number of patients treated over time. The SVR results confirm previous research showing HCV resolution in >95% of treated patients. Israel initiated and executed a comprehensive HCV screening and treatment program on a national scale in just 2 years, aligning with WHO’s global initiative for HCV elimination. By implementing clear criteria for identifying targeted at-risk persons, mandating their screening, and financially supporting and adequately using existing HMOs’ EMR infrastructure, almost two thirds of the at-risk population was screened and treated in less than 2 years. Nonetheless, in view of the abovementioned limitations, HCV likely will not be eliminated in Israel by 2030, but HCV prevalence will substantially decrease.

Appendix Additional information on an economic analysis of a national program for hepatitis C elimination, Israel, 2023.
