# Supplementary material for: Economic Analysis of National Program for Hepatitis C Elimination, Israel, 2023
Source: Emerg Infect Dis. 2024 Oct;30(10):2070–8. doi: 10.3201/eid3010.240210 (PMC11431911; doi:10.3201/eid3010.240210)
Supplement: Appendix — Additional information on an economic analysis of a national program for hepatitis C elimination, Israel, 2023. [file 24-0210-Techapp-s1.pdf]

Article DOI: https://doi.org/10.3201/eid3010.240210

EID cannot ensure accessibility for supplementary materials supplied by authors. Readers who have difficulty accessing supplementary content should contact the authors for assistance.

Economic Analysis of National Program for Hepatitis C Elimination, Israel, 2023

Appendix

Appendix Table. Budgeting HCV treatment on the Israeli health basket over the years*

Year	Medications	Indications	Cost, million NIS
1995	Interferon alfa		No additional cost
1999	Ribavirin		15
2005	Pegylated interferon alfa	Naïve patients	30.596
2009	Pegylated interferon alfa	Patients with progression post PEGylated interferon	9.521
2012	Boceprevir, Telaprevir	Genotype 1 with fibrosis stage F2–F4	43.972
	Fibroteset and fibroscan for fibrosis staging and viral load		
2015	Paritaprevir + Ritonavir + Ombitasvir, Dasabuvir	Genotype 1 with fibrosis stage F3–F4	74.931
2016	Paritaprevir + Ritonavir + Ombitasvir, Dasabuvir	Genotype 1 with fibrosis stage F3–F4	25
	Daclatasvir, Sofosbuvir	Genotype 2,3,4 with fibrosis stages F3–F4	56.269
	Dasabuvir	Genotype 4 with fibrosis stages F3–F4	
	Fibrotest and fibroscan for fibrosis assessment and follow-up. Genotyping test		5.58
2017	Grazoprevir + Elbasvir,<br>Sofosbuvir + Ledipasvir,<br>Sofosbuvir + Velpatasvir,<br>Ombitasvir + Paritaprevir + Ritonavir, Dasabuvir	For genotype 1 or 4, regardless of fibrosis stage, or together with: HIV, HBV, liver transplantation, HCV recurrence, extra-hepatic manifestations	No additional cost
2018	Grazoprevir + Elbasvir<br>Sofosbuvir + Ledipasvir<br>Dasabuvir, Paritaprevir + Ritonavir + Ombitasvir<br>Paritaprevir + Ritonavir + Ombitasvir<br>Sofosbuvir + Velpatasvir<br>Sofosbuvir + Velpatasvir + Voxilaprevir<br>Glecaprevir + Pibrentasvir	Genotype 1,4 with fibrosis stages F0–F1<br>Genotype 2,3 with fibrosis stages F0-F2<br>DAA treatment failure	No additional cost
Total cost			260.869

*DAA, direct-acting antiviral; NIS, New Israeli Shekel.
